# TOPS: a versatile software tool for statistical analysis and visualization of combinatorial gene-gene and gene-drug interaction screens

**DOI:** 10.1186/1471-2105-15-98

**Published:** 2014-04-08

**Authors:** Markus K Muellner, Gerhard Duernberger, Florian Ganglberger, Claudia Kerzendorfer, Iris Z Uras, Andreas Schoenegger, Klaudia Bagienski, Jacques Colinge, Sebastian MB Nijman

**Affiliations:** 1 Research Center for Molecular Medicine of the Austrian Academy of Sciences (CeMM), Vienna, Austria; 2 Current address: The Research Institute of Molecular Pathology (IMP), Vienna, Austria

**Keywords:** Double perturbation screens, Synthetic lethality, Functional genetics, Massive parallel sequencing, Luminex xMAP, Drug screens, Synergy, Epistasis

## Abstract

**Background:**

Measuring the impact of combinations of genetic or chemical perturbations on cellular fitness, sometimes referred to as synthetic lethal screening, is a powerful method for obtaining novel insights into gene function and drug action. Especially when performed at large scales, gene-gene or gene-drug interaction screens can reveal complex genetic interactions or drug mechanism of action or even identify novel therapeutics for the treatment of diseases.

The result of such large-scale screen results can be represented as a matrix with a numeric score indicating the cellular fitness (e.g. viability or doubling time) for each double perturbation. In a typical screen, the majority of combinations do not impact the cellular fitness. Thus, it is critical to first discern true "hits" from noise. Subsequent data exploration and visualization methods can assist to extract meaningful biological information from the data. However, despite the increasing interest in combination perturbation screens, no user friendly open-source program exists that combines statistical analysis, data exploration tools and visualization.

**Results:**

We developed TOPS (Tool for Combination Perturbation Screen Analysis), a Java and R-based software tool with a simple graphical user interface that allows the user to import, analyze, filter and plot data from double perturbation screens as well as other compatible data. TOPS was designed in a modular fashion to allow the user to add alternative importers for data formats or custom analysis scripts not covered by the original release.

We demonstrate the utility of TOPS on two datasets derived from functional genetic screens using different methods. Dataset 1 is a gene-drug interaction screen and is based on Luminex xMAP technology. Dataset 2 is a gene-gene short hairpin (sh)RNAi screen exploring the interactions between deubiquitinating enzymes and a number of prominent oncogenes using massive parallel sequencing (MPS).

**Conclusions:**

TOPS provides the benchtop scientist with a free toolset to analyze, filter and visualize data from functional genomic gene-gene and gene-drug interaction screens with a flexible interface to accommodate different technologies and analysis algorithms in addition to those already provided here. TOPS is freely available for academic and non-academic users and is released as open source.

## Background

Genes operate in complex cellular networks, and elucidating this connectivity is critical for understanding normal physiology and disease. Functional genomic screens that combine two perturbations have been used with great success to uncover such genetic interactions, and also to reveal mechanisms of drug action [1,2]. This is generally done by perturbing a biological system, most often a cell, in a defined manner i.e. by overexpression or knockdown of a gene or by addition of a drug and subsequently administering a second set of perturbations and analyzing the system for non-additive readout (i.e. synergistic/antagonistic or synthetic lethality/synthetic rescue) [3].

These perturbation experiments have traditionally been performed in model organisms such as yeast where genetic manipulation is relatively easy compared to mammalian cells [2]. Now, with the advent of technologies including RNA interference (RNAi) and the recent emergence of nuclease-based genome engineering, human cells can also be used to perform functional genomic screens. Cellular fitness, often represented by cell number, division rate or death, is used as a proxy for many of these screens since it is a parameter that can be measured with relative ease. This proxy is also of particular relevance in case genetic interactions are sought as potential anti-cancer therapies. Here, the aim is to kill only cells carrying cancer mutations by targeting an interacting gene which is in synthetic lethal configuration to the mutation. This can be achieved by targeting this second gene product with a small molecule inhibitor or antibody. The non-cancer cells would remain unaffected from this therapy as inhibiting only one interaction partner will not lead to lethality. Indeed, many cancer mutations can be tested systematically against drugs to uncover selective resistance/sensitivity mechanisms, or against small interfering RNAs to find potential new drug targets [4-6].

A pairwise wise interaction screen yields a matrix of interaction (or fitness-) scores. For two non-overlapping sets of perturbations *A* and *B* the number of measurements and statistical tests *T* required to evaluate all unique pairwise combinations is given by:

$$T=\left|A\right|\times \left|B\right|$$

In practice, the number of data points typically generated is further increased to T x n by the requirement of n replicate measurements. Thus, a screen of, for example, 100 genes against 100 drugs measured in quadruplicate requires 40,000 measurements. A Dataset of this size is usually beyond the limits of what can be conveniently analyzed without the use of dedicated statistics software.

To generate these large datasets, multiplexing strategies greatly improve the throughput of the experiment, by combining genetic perturbations in a single well (multiplexing drug treatments is typically not feasible). A convenient multiplexing strategy is to introduce DNA barcodes into the genome of mutant cell lines such that each genetic perturbation corresponds to a single barcode sequence. In this scenario DNA barcodes act both as unique identifiers for each genetic perturbation and as a proxy for cell number/fitness. Barcodes can be encoded by short hairpin (sh)RNA sequences themselves or introduced into the genome as non-transcribed DNA sequences using lentiviral vectors [7,8]. Practically, a set of barcoded cell lines (A) can then be pooled together and divided into aliquots that are then screened against the second set (B) of perturbations.

**Figure 1:**
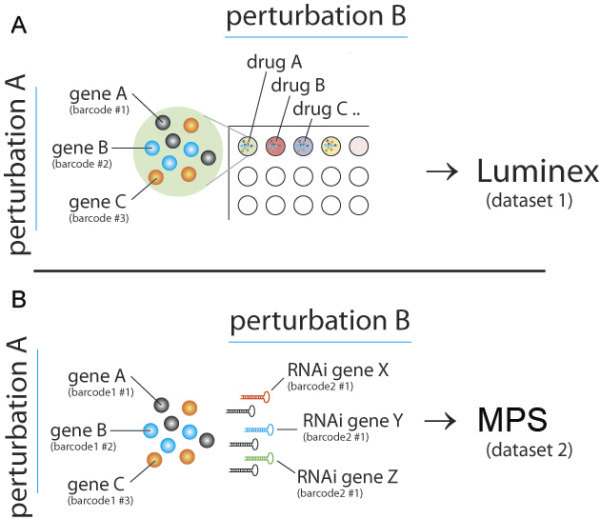
**Overview of the two discussed multiplexing technologies used for gene-drug and gene-gene interaction studies. (A)** Luminex multiplexing for a gene-drug interaction screen. The gene perturbation is multiplexed and encoded as a DNA barcode that can be hybridized to Luminex beads and quantified. Drugs are not multiplexed. **(B)** MPS double multiplexing screen for gene-gene interactions. The first (genetic) perturbation can be multiplexed as a Illumina index barcode, and the second perturbation is identified by sequencing the hairpin cassette, which acts as a second DNA barcode.

In our laboratory we have generated multiplexed gene-gene and gene-drug interaction data using two distinct technologies [Figure 1]:

(1) Luminex xMAP – a hybridization based multiplexing technology that allows up to 500 measurements in a single sample. Here, DNA barcoded panels consisting of genetically modified cell lines (i.e. expressing an oncogene or knockdown of a tumor suppressor gene) and an unmodified control from the same genetic background are pooled in a single well of a multiwell plate (genetic perturbation, set A) and treated with either a drug or a second genetic perturbation (drug perturbation, set B). In this case only perturbation set A is multiplexed.

(2) Massive Parallel Sequencing (MPS). In this case, a panel of non-barcoded cell lines carrying perturbation set A from (1) are infected with a library of lentiviral shRNA vectors (genetic perturbation, set B). Here, the shRNA sequence that integrate into the genome serves both as perturbation and as DNA barcode. Further multiplexing can be achieved by indexing the second perturbation in a single sequencing run. For example, a MPS run yielding 10^8^ aligned reads on average allows 100 cell lines to be screened against 100 drugs at a reasonable average sequencing depth of 10,000 reads per DNA barcode.

In both cases the abundance of DNA barcodes amplified from the genomic DNA is measured after a period of exponential cell growth and used as a score for cellular fitness. To deconvolute, analyze and visualize this type of data in a modular and intuitive manner we established TOPS.

## Implementation

### Programming Language, external software and system requirements

TOPS was developed as a graphical user interface for an analysis pipeline that we originally developed in R. In order to make these R scripts accessible to non-expert users, in particular scientists with little background in scripting languages, we took advantage of the R package rJava ( http://www.rforge.net/rJava/) to run the scripts from a Java based interface. TOPS can therefore run its R scripts on all platforms for which rJava is available (currently MacOS, Linux and Windows).

**Figure 2:**
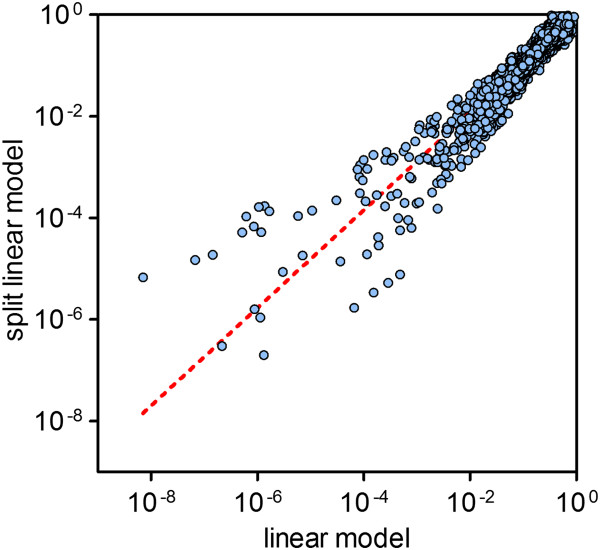
**Correlation of p-values for linear and split linear model algorithms.** Splitting the data set into smaller subsets and normalizing as well as analyzing each of them separately only introduces minor deviations in the resulting candidate hits (i.e. 39 of the top 50 hits in one method are among the respective top 50 of the other method).

The hardware requirements to run TOPS depend mostly on the volume of data to be analyzed and the algorithm to be used. We have developed and tested TOPS on two independent datasets derived from different experimental approaches, and on a standard office computer with a 2.4 GHz dual core processor and 2.5 GB of RAM. We note that for larger datasets the linear model (linearModel.R) algorithm provided with this release requires more memory than is usually available on an office computer. Therefore, we have provided an alternative that splits the analysis in smaller parts (linearModel_split.R) and later assembles the results. This modification necessitated an adjustment in the data normalization [Figure 2].

Output from the R interface, including and error messages, is stored in text files for troubleshooting. The overall progress of a session can be monitored and is logged separately.

### User skills and installation

Installation of TOPS consists of unpacking the zipped file into a directory. For TOPS to run, Java, R and the package rJava are must also be installed. Upon first launch, definition of the paths in which R and rJava are located may be required. The most common locations for MacOS, Linux and Windows are checked automatically and the user will be prompted only if the libraries cannot be found. For convenience, alternative locations to be checked in this manner can be added in libPaths.txt in the TOPS directory.

TOPS uses a single semicolon separated values (CSV) data input file. This file has to be generated by the user and can for instance be exported from a Microsoft Office Excel spreadsheet. Alternatively the example datasets can be used for testing. To use TOPS no scripting skills are necessary.

## Results and discussion

### Example datasets provided

TOPS comes with two datasets to test the different procedures. Thus, users can explore the software without having to generate original data.

#### Example dataset 1: a gene-drug interaction screen in breast cancer

To simulate oncogenic events in breast cancer we created a panel of 70 different isogenic cell lines ectopically expressing oncogenes or knocking down tumor suppressor genes commonly found in breast cancer. Findings based on this dataset have been published previously [7].

For multiplexing purposes, each cell line was infected with a vector introducing a genetic barcode (xMAP tag) into the genomic DNA. The cell lines were then pooled, distributed among multi-well plates and treated with 87 individual drugs. After a period of exponential growth, genomic DNA was harvested and barcodes were quantified using Luminex xMAP beads. Raw bead data was converted to median measurements per interaction by calculating the truncated mean. The resulting data is in the format ‘Cell line (name)’, ‘Drug (name)’, ‘Barcode score (numerical)’. The barcode score is a measure of the relative fitness as it is an estimate of the total number of cells for each isogenic cell line under the drug treatment condition.

#### Example dataset 2: a gene-gene interactions screen in breast cancer

We screened 27 isogenic cell lines expressing oncogenes against a library consisting of approximately 400 shRNA vectors covering 80 deubiquitinating enzymes (DUBs). DUBs represent an emerging class of cancer targets involved in many different cellular processes with therapeutic potential. After a period of exponential growth cells were harvested and genomic DNA was extracted in order to PCR-amplify the shRNA barcode cassettes. During the amplification process primers with 60 unique barcode sequences corresponding to the 27 cell lines in 4 replicates were used as described in [9]. The tagged PCR samples were then pooled, a sequencing library was generated and the samples were sequenced on two lanes (50 basepairs) of a Hiseq 2000 instrument (Illumina). The MPS raw data were converted to FASTQ format and split according to the indexing PCR primers with a custom Python script. Each sample’s NGS reads were aligned to the shRNA sequences using shALIGN [10] allowing for zero, one, and two mismatches. The output CSV file is in the format: ‘Cell line (name)’, ‘shRNA (name)’, ‘number of reads’. Number of reads corresponds to shRNA abundance and this is the readout for relative fitness.

#### Importing data

A typical input file consists of five columns with the following information:

perturbation A; perturbation B; fitness score; replicate 1; replicate 2

In the provided example dataset 1, perturbation A corresponds to the gene (i.e. overexpression or knockdown) and perturbation B is a drug treatment. For dataset 2, perturbation B is an shRNA vector targeting a specific DUB and perturbation A is the gene. Fitness score represents the barcode abundance in these examples. Replicates 1 and 2 designate if measurements are replicates of each other. Replicate 1 identifies multiple perturbations towards the same outcome (i.e. multiple different hairpins for one gene, multiple vectors encoding the same cDNA) while replicate 2 identifies the replicate number for a given A-B combination.

TOPS has custom importers covering both types of files from the example datasets. These importers convert the data coming from Luminex or MPS to the input file format.

Entries with non-unique identifiers for perturbation A and perturbation B are automatically interpreted to be replicate measurements by the importers.

#### Pre-analysis filtering

**Figure 3:**
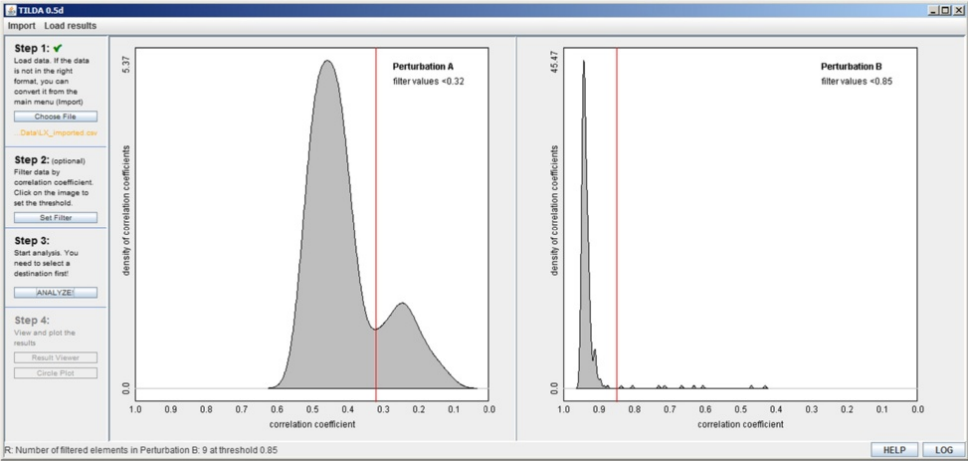
**Pre-analysis filtering for both sets of perturbations.** Spearman correlations are plotted as a histogram for both sets of perturbations. Noisy perturbations can be removed before normalization by changing the cutoff slider according to the desired stringency.

We implemented a filtering method to allow the user to detect and remove bad replicates or samples before running the analysis as this can result in false positive hits. This filtering is based on the assumption that the frequency of gene-gene or gene-drug interactions is low among all possible combinations [11]. Therefore noisy perturbations can be identified by how well they correlate with others. For each perturbation a pairwise Spearman correlation is calculated against all other perturbations in the dataset. A poor correlation then indicates a technically noisy sample that the user may wish to exclude from further analysis. We perform this correlation for both A and B perturbations and correlation cutoffs can be chosen for each individually [Figure 3].

#### Analysis algorithms

TOPS is supplied with two analysis models that are well suited for interaction data of the type that is supplied in the example datasets. However, custom analysis methods can be conveniently plugged into TOPS. The first method is based on a multiple linear regression model and the second one is based on the Mann-Whitney U Test.

#### Statistical analysis: linear model

Linear models have been used previously to normalize [12,13] and analyze [14,15] high-throughput measurements. Accordingly, our analysis is composed of two consecutive linear models: the first one is used for data normalization; the second one identifies outliers that represent "hits" i.e. potentially biologically meaningful interactions that warrant further study.

Data normalization relies on the assumption that relative fitness values *intensity*_*i*_ as determined by barcode quantification (via MPS or Luminex) are constituted of four components:

1) the systematic effect of perturbation A on fitness

2) the systematic effect of perturbation B on fitness

3) the signal that is due to the (synergistic) combination of perturbation A and B consistent across biological replicates, which is the signal of biological interest

4) noise caused by biological and technical variability between the replicates.

These four components can be modeled as:

$$\text{intensit}y_{i}=\beta_{A}\;A+\beta_{B}\;B+s_{i}$$

where *intensity*_*i*_ represents the measured fitness score, *β*_*A*_, *β*_*B*_ are regression coefficients, *A*, *B* are categorical variables representing perturbations A and B respectively, and *s*_*i*_ is a remaining signal including both noise (biological and technical) and a potential synergistic contribution of A and B perturbations. The model is fit to the experimental data to estimate the first two components from the fitness score corresponding to the systematic, non-synergistic perturbations induced by A and B. The linear model was fit by robust regression using an M estimator (function rlm in R library "MASS") to avoid influence of outliers.

The second linear model further dissects the component *s*_*i*_ to identify potential synergy. Since we want to analyze the effect of each perturbation on the other one, *s*_*i*_ is dissected twice by fixing either the perturbation A or B first. Namely, fixing A first, we can reduce *s*_*i*_ to the signal observed for each successive value of A, which we denote by *s*_*i*|*A*_. On this reduced data, the effect of perturbation B is estimated by the model:

$$\forall A:s_{i|A}=\beta_{B|A}B+\varepsilon$$

where *A*, *B* are categorical variables as above, *β*_*B*|*A*_ the regression coefficient, and *ε* a term capturing the contribution of noise. The same procedure is applied when perturbation B is fixed first:

$$\forall B:s_{i|B}=\beta_{A|B}A+\varepsilon$$

The coefficients of these linear models (*β*_*A*|*B*_ and *β*_*B*|*A*_) capture the effect of a perturbation within the context of a specific other perturbation. The coefficients combine the signal of replicate experiments and estimate the magnitude of the biological effect thanks to the noise component *ε*.

The ‘rlm’ function of the R ‘MASS’ library is employed to compute a robust estimate of the coefficients of the linear model. This function also offers the possibility to calculate the statistical significance of each coefficient by performing a one-sided t-test (*β*_*A*|*B*_ ≠ 0 or *β*_*B*|*A*_ ≠ 0). For small sample sizes this test can suffer from false positives due to underestimating variance as described in Axelsson et al. [16].

As both linear models are applied after log transformation of the input data these additive coefficients reflect modeling a multiplicative effect of the fitness response of the pairwise fitness effects *β*_*A*_and *β*_*B*_[17,18].

In the case of the sLM split linear model aimed at reducing computation time and memory usage, the dataset is subdivided into groups of 50 perturbations (A and B), resulting in less data points available for the t-test, hence the reduced statistical power.

#### Statistical analysis: Mann-Whitney U-test

The second method we implemented to analyze fitness score data is based on the *Mann-Whitney U-test*, a more robust alternative to the *Student’s t-test* that does not assume a normal distribution. For this analysis a distinct normalization procedure is employed.

First, numerical data was arranged in a matrix with rows and columns representing perturbations A and B, respectively. Next, we calculate a normalized value for each gene-gene or gene-drug "interaction" in the matrix as:

$$m_{\text{ij}}=\frac{A_{\text{ij}}B_{\text{ij}}}{\text{median}\left(A_{j}\right)\text{median}\left(B_{i}\right)}$$

Here, *i* and *j* represent row and column positions in the matrix, and *A*_*j*_ and *B*_*i*_ represent sets containing all values for row *j* and column *i,* respectively. Thus, each value in a row is divided by the corresponding row median, and each value in a column by the column median across the matrix. Each fitness score in the matrix is subsequently log_10_ transformed.

Next, the algorithm performs two-sided tests to calculate p-values. The model requires at least 3 replicates per interaction to perform this test. As with the linear method algorithm we calculate two p-values for each interaction, corresponding to perturbation set A or B.

#### Combining p-values

**Figure 4:**
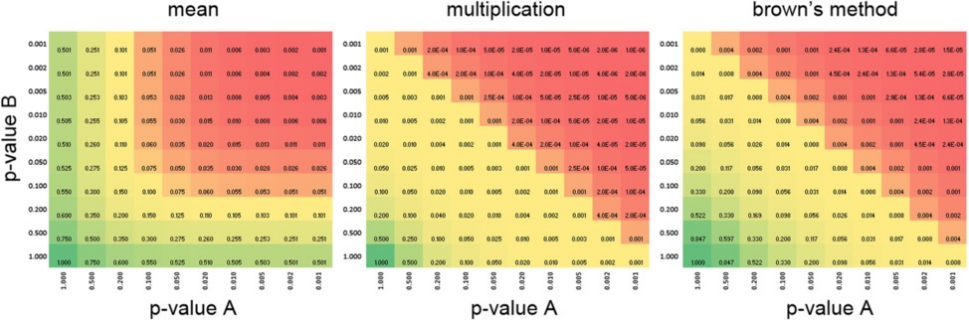
**Comparison of different methods for combining scores.** Heatmap of the overall score out of the two individual p-values by either mean (left), multiplication (middle) or Brown’s Method (right).

Both methods of analysis presented above calculate p-values in either direction of the interaction matrix. This means that either the distribution of A’s or B’s for a given A-B interaction matrix is used to derive the significance score. The distributions of A’s and B’s are likely to be different, depending on the assay technology used to generate the data. Therefore a pvalue derived from one distribution (pvalue A) is not necessarily equal to the pvalue derived from the second distribution (pvalue B) for the same A-B interaction. To obtain an overall score to rank hits, we tested three methods to combine p-values: (1) calculating the average of both values, (2) multiplication of both values with each other, and (3) Brown’s Method [19], a modification of Fisher’s method for combining dependent p-values [Figure 4]. Taking the average of two p-values strongly penalized fitness scores just below the significance level, potentially resulting in more false negatives while Brown’s Method rewarded combinations where both p-values are close to significant. Multiplication of both p-values gave comparable but slightly more extreme results than Brown’s. We therefore selected Brown’s Method to calculate combined p-values as a conservative middle ground between the other two more extreme methods.

#### Importing analyzed data into the "results browser" and generating plots

Once data has been analyzed by either method, the results of the analysis are stored in a text file in the following format:

perturbation A; perturbation B; relative magnitude A; relative magnitude B; absolute magnitude A; absolute magnitude B; p-value A; p-value B; combined p-value by Brown’s Method.

Where perturbation A and B are the identifiers for the tested perturbations, relative magnitude A and B gives an indication of the ratio of treatment versus the median of all treatments (control), absolute magnitude A and B are the raw signals from the assay and the p-values are calculated as discussed previously. Given this format, other datasets not generated with our analysis pipeline can also be imported, filtered and plotted using TOPS.

#### Post-analysis filtering

**Figure 5:**
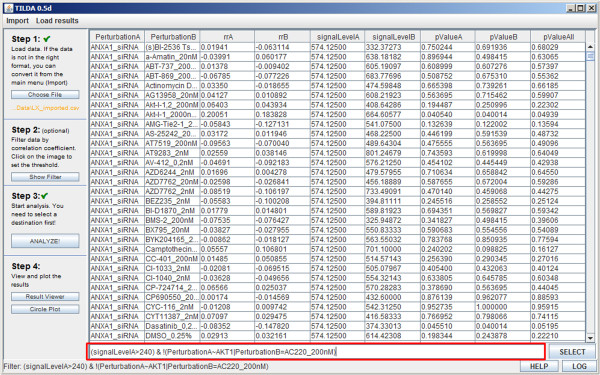
**Screenshot of the results browser.** Data from the analysis can be filtered by any of the parameters displayed in the header of the table. Filtering strings can be entered in the text field below the results table and data can be sorted by clicking on a column name.

After the analysis is complete, the results are displayed in a table in the results browser [Figure 5] and can be sorted ascending or descending by clicking on the column headers. Here the user can sort and filter the obtained results before visualization by entering a string of filtering criteria. These criteria can be connected with the AND (&) or OR (|) operator and also allows for partial matches using the tilde (~) sign or exclusion using a logical NOT (!). Details on filtering strings can be found in TOPS’s help files. The optimal filtering parameters vary with the technology used to obtain the data. In our experience, the mean Luminex signal (xMAP) and mean number of reads (MPS) are reasonable parameters to use for filtering out false positives. Removing the bottom 5-10% of signals sorted by these parameters gave a satisfactory reduction in noise.

#### User modules

**Figure 6:**
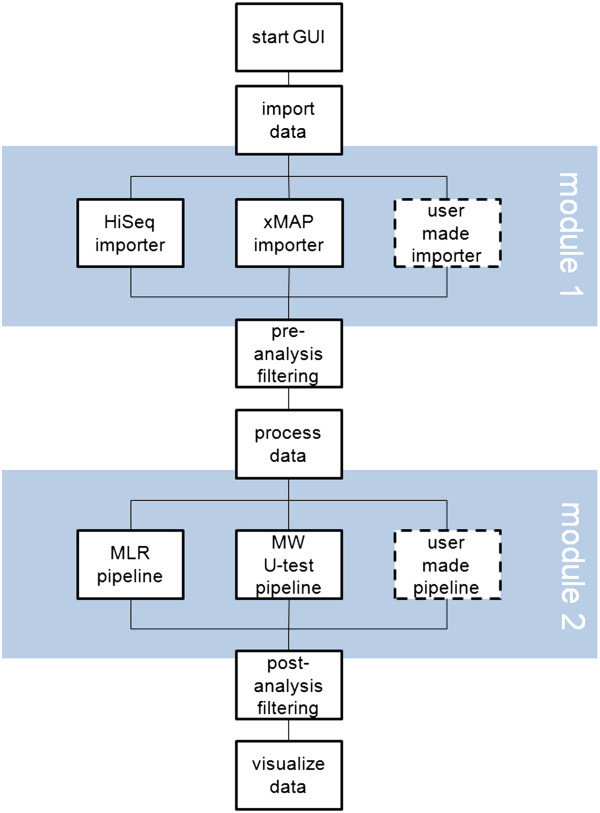
**Basic layout of the analysis process in TOPS.** TOPS has been written in order to be flexible with regard to user modules. User made importers can be written in Java and integrated with the GUI while custom analysis algorithms can be supplied as R scripts.

TOPS has been designed in a way that allows future users to easily write new importers in java to support other input data formats. We have also implemented the possibility to run user-written R-code for the data analysis which can then be called from within TOPS and fed into the data filtering and visualization [Figure 6]. The R scripts provided with this release can be used as templates to write custom modules as required.

#### Comparison of performance of the analysis models

In order to compare the performance of all three models (linear, split-linear, and Mann-Whitney U-test) we generated a randomized dataset with a distribution based on dataset 1. In this set we generated true positive hits with an average signal of 0.5, 0.7 (reduced fitness) 1.3 and 1.5 fold (increased fitness) compared to the average of true negatives.

**Figure 7:**
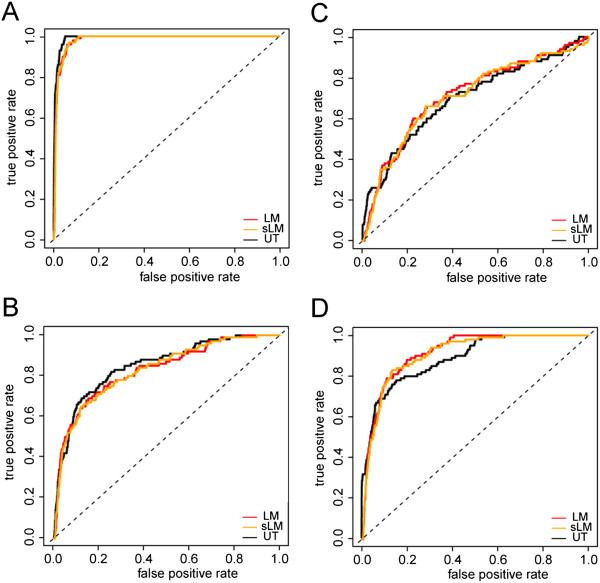
**Receiver operating characteristics for the analysis algorithms.** ROC curves are derived from a random 100x100 perturbations dataset in quadruplicate modeled after dataset 1 where 100 true positives were introduced and defined as either 0.5 **(A)**, 0.7 **(B)**, 1.3 **(C)** or 1.5 **(D)** times that of the true negatives.

**Figure 8:**
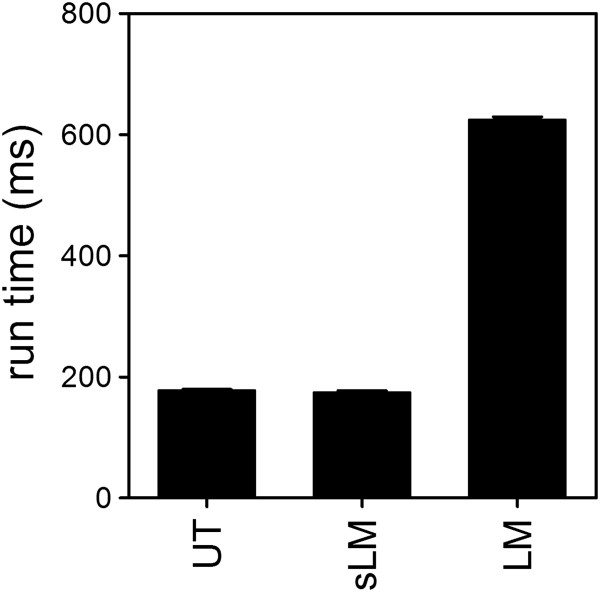
**Runtime performance of the three algorithms.** The U-Test (UT), split linear model (sLM) or linear model (LM) algorithms were tested for runtime performance analyzing a set of 200×200 interactions.

To estimate false positive and false negatives, we used this dataset to determine receiver operating characteristics (ROC) for all three algorithms [Figure 7]. Overall, all algorithms show comparable characteristics with the U-Test performing slightly better at lower-fitness hits, and the linear models being more sensitive for increased fitness hits. In general, decreased fitness hits were more readily detected by our algorithms due to the non-normal distribution of the data. In terms of run-time performance the U-Test and Split Linear Model algorithm are practically identical while the Linear Model algorithm is slightly more computationally costly [Figure 8].

For dataset 1, interactions have also been validated experimentally. Comparing the three algorithms in terms of the verified true positives they can identify from a ranked list of the top 50 hits after filtering as described in [7] shows comparable performance [Table 1] and an acceptable true/false positive rate overall compared to other high-throughput screens [20].

**Table 1:** Experimentally validated true and false positive hits from dataset 1

**Model (pvalue)**	**True positives**	**False positives**	**Unverified**
LM (pvA)	8	2	40
LM (pvB)	1	1	48
LM (pvAll)	6	2	42
sLM (pvA)	8	2	40
sLM (pvB)	2	0	48
sLM (pvAll)	8	1	41
UT (pvA)	7	2	41
UT (pvB)	4	2	44
UT (pvAll)	6	2	42

The top 50 interactions from a ranked hit list are categorized by experimentally validated as true or false positive. Hits that were not experimentally tested are indicated as "unverified". The true and false positive rates for the linear model (LM), split linear model (sLM) or U-Test (UT) algorithms for either p-value A (pvA), p-value B (pvB) or the combined p-value (pvAll) are shown.

**Figure 9:**
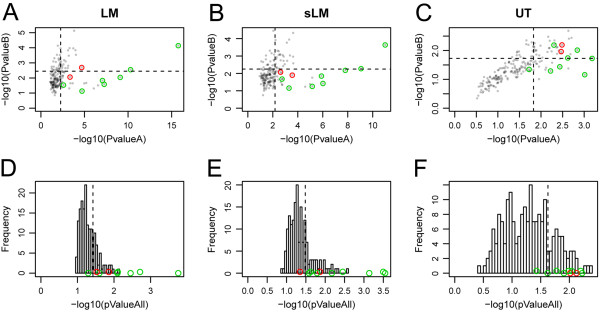
**Separation of successfully versus failed validations of selected hits.** The cut-off for the top 50 hits are indicated by dashed lines. **(A-C)** Position of validations in the P-value **A** and **B** space (log-scale). **(D-F)** Distribution of all the combined P-values (**A** and **B** combined according to Brown’s method) with positions of validated hits. Experimentally validated true positives are indicated in green, false positive hits in red.

In [Figure 9A-C] we show a scatter plot of P-values for perturbations A and B with the successful and failed validation indicated. We can observe an increasingly better spatial separation comparing the U-test with sLM and LM models, which could be for instance be captured by principal component analysis. However, since the localization of successful versus failed validation data points does not follow a simple pattern according to the original coordinates provided by the two P-values and is likely to be different for distinct datasets, this would necessitate to have available such a validation dataset to train a classifier performing better than simple P-value-based thresholds. As a generic tool TOPS does not offer this functionality but users might consider implementing it since we release the code of the system as open source. Combining the P-values for perturbations A and B does not have a negative effect on discriminative power but allows to provide a single interaction score [Figure 9D-F].

#### Data visualization

**Figure 10:**
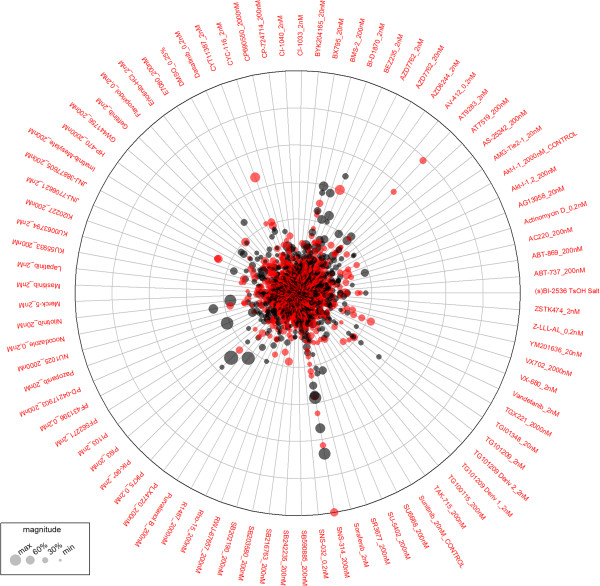
**TOPS’s graphical output.** Results of the analysis are displayed on a circular bubble plot with the color encoding the direction (reduced fitness, red or increased fitness, black) of the interaction, distance from the center indicates score/pvalue and bubble size corresponds to the magnitude of the measured effect. Labels at the circumference indicate the perturbation identifier, in this example drugs.

Data from gene-gene or gene-drug interaction screens can be difficult to inspect visually since the number of hits is comparatively small to the number of interactions. Traditional plotting methods from gene expression studies like heatmaps do not perform well in this setting. We therefore designed a circular bubble plot to display the data. An axis for every condition B is drawn on which the scores/p-values for the interactions with each condition A are plotted. The axes are arranged in a radial fashion such that the least significant hits are in the center of the plot and the most significant interaction scores are on the circumference of the circle. This allows the user to quickly spot outliers and problematic/noisy perturbations. In addition the magnitude of the effect can be encoded in the size of each dot, allowing the user to identify hits that are both significant and show a reasonable effect size. The direction of the interaction (increased or decreased fitness as compared to control) is encoded by different colors [Figure 10]. The identifiers of one set of perturbations is plotted at the circumference of the circleplot. The second perturbation can be identified by hovering the mouse over a datapoint in the plot. TOPS’ plots can be exported as bitmaps in PNG format or as vector graphics as a PDF file.

## Conclusions

We present a user-friendly software package to analyze and visualize interaction data from functional genomic dual perturbation screens. Although multiple tools for analyzing cell viability screens are available [16,21-24] these have their limitations by either being based on commercial software (mostly MATLAB) or requiring command line skills. TOPS incorporates statistical models designed for the analysis of pairwise interactions of larger gene/drug sets. Furthermore it is fully based on free software and provides a graphical user interface. The software is easily accessible and offers a powerful analysis tool for the benchtop scientist while being expandable enough to be attractive to users who would like to run their own analysis methods. Importantly, not only Luminex xMAP and Sequencing data can be analyzed with the presented methods but in principle any data from other technologies can be imported as long as the data can be reduced to a "perturbation A", "perturbation B", "score" format and true positives are relatively rare in comparison to true negatives which is a necessity due to the nature of normalization of the in-built analysis. We have included two analysis pipelines based on different methods to demonstrate the versatility of TOPS. We have also included two importers for Luminex xMAP data and for pre-processed screening data that makes these two technologies particularly easy to use with the software.

## Availability and requirements

**Project name:** TOPS.

**Project home page:**https://sourceforge.net/p/topscemm/wiki/Home/.

**Operating system(s):** Win32/OSX/Linux.

**Programming language:** Java and R.

**Other requirements:** Java 6 or newer. R 2.14 or newer.

**License:** Creative Commons Attribution ShareAlike License V3.0.

**Any restrictions to use by non-academics:** Only those imposed by the license.

## Competing interests

The authors declare that they have no competing interests.

## Authors’ contributions

MKM and FG designed and FG wrote the code for TOPS and the help files. CK and MKM created the datasets. IU, MKM and CK performed wetlab validation experiments. GD and JC designed and GD wrote the Linear Model based algorithm. FG and MKM designed and wrote the U-test based algorithm. KB and AS helped with processing the sequencing data. GD produced Figure 8, MKM produced the remaining figures and together with SN wrote the manuscript with the help and contributions from the other authors. SN, MKM and JC co-supervised the project. All authors read and approved the final manuscript.
